# Dataset on recombinant expression of an ancient chitinase gene from different species of *Leishmania* parasites in bacteria and in *Spodoptera frugiperda* cells using baculovirus

**DOI:** 10.1016/j.dib.2020.106259

**Published:** 2020-09-02

**Authors:** Aline Diniz Cabral, Felipe Baena Garcia, Rodrigo Buzinaro Suzuki, Tanil Lacerda Góis Filho, Renata Torres da Costa, Ligia Marinho Pereira Vasconcelos, Edmar Silva Santos, Márcia Aparecida Sperança

**Affiliations:** a Center for Natural and Human Sciences, Universidade Federal do ABC, Rua Arcturus, 03 - Bloco Delta, Sala 226, Laboratório 107, 09606-070 São Bernardo do Campo, SP, Brazil; b Department of Parasitology, Marília Medical School, 17519-030 Marília, SP, Brasil; c School of Medicine, University of Marilia, 17.525-902 Marília, SP, Brazil

**Keywords:** *Leishmania sp*, Chitinase, Baculoviruses, Sf9 insect cell, Recombinant protein

## Abstract

The data presented here is related to negative results obtained with the recombinant expression of chitinase from four species of *Leishmania* parasites in two expression systems, performed in order to investigate the molecular characteristics of the *Leishmania* chitinase and its possible application in leishmaniasis diagnosis. Thus, heterologous *Leishmania sp* chitinase proteins were expressed in bacteria using the prokaryotic expression vector pET28a and *Escherichia coli* Mach-T1, and in *Spodoptera frugiperda* (Sf9) insect cells, using the eukaryotic *bac-to-bac* expression system (Thermo Fisher Scientific) to produce recombinant baculoviruses to infect Sf9. Biochemical and cellular analysis of the various recombinant forms of the *Leishmania sp* chitinase produced in prokaryotic and eukaryotic expression systems were performed through SDS-PAGE and Western blotting. Chitinase produced and purified from bacteria presented low yield and formed inactive aggregates. Heterologous chitinase obtained after infection of Sf9 insect cells with all the four *Leishmania* species recombinant baculoviruses presented high yield of insoluble proteins. Dot-blot serological tests presented inconclusive results against the recombinant *Leishmania* sp chitinases produced in both expression systems. The experiments described in this paper can help researchers to avoid errors when choosing a recombinant expression systems to produce *Leishmania* parasites proteins for biotechnological purposes.

## Specifications Table

**Table:** 

Subject	Biochemistry, genetics, and molecular biology
Specific subject area	Expression and production of recombinant protein in prokaryotes and eukaryotes
Type of data	Table Image Graph Figure
How data were acquired	Nucleic acid electrophoresis, sodium dodecyl sulphate polyacrylamide gel electrophoresis (SDS-PAGE), Western blotting, spectrometry Instruments: horizontal and vertical electrophoresis apparatus from BioRad®, UV transiluminator and digital photographic camera
Data format	Raw and analysed data
Parameters for data collection	Data were collected using nucleic acid and proteins electrophoresis through agarose gels, SDS-PAGE and Western blotting.
Description of data collection	Chitinase encoding gene without signal peptide from different *Leishmania* species were cloned into baculovirus DNA and into the pET28a plasmid, which were used to infect *S. frugiperda* Sf9 insect cells and MachT1 bacteria, respectively. Recombinant proteins were analysed through SDS-PAGE and Western blotting with antibody against His-tag and alkaline phosphatase colorimetric detection. The sequence of chitinase without signal peptide encoding gene from *L.eishmania amazonensis* was deposited in Genbank [MG869127], URL: https://www.ncbi.nlm.nih.gov/nuccore/MG869127.1?report==genbank&log$=seqview
Data source location	Centro de Ciências Naturais e Humanas – Universidade Federal do ABC, São Bernardo do Campo, São Paulo, Brazil, -23.67662000; -46.56263300, data collection from January 2014 to December 2017.
Data accessibility	All data are presented with the article

## Value of the Data

• The chitinase protein is unique among kinetoplastida. It is present in the *Leishmania* genus and not in the *Trypanosoma* genus*,* being an excellent molecule to be used in specific diagnostic serological tests for *Leishmania* species.
• The data presented can benefit all researchers working on recombinant protein production from *Leishmania* species for biological function, molecular structure investigation, and biotechnological applications.
• Considering the biotechnological potential of *Leishmania* chitinase, the data described in this paper can help in the choice of others expression systems, that might produce a higher amount of the correct folded protein.

## 1 Data Description

Protozoan parasites of the *Leishmania* genus are the causative agent of the leishmaniases that are transmitted among humans, domestic dogs, and wild animal hosts by insect vectors of the Psycodidae Family (sandflies), as well as the Phlebotomus (Old World) and *Lutzomyia* genus (New World - the Americas) [1]. All fifty-three *Leishmania* species are divided into five groups, the subgenera *Leishmania, Viannia, Sauroleishmania, Mundini*, and *Paraleishmania*. From these, 20 can cause disease in humans [1]. The *Leishmania* subgenus is involved in visceral leishmaniasis in the Old World and it causes tegumentar leishmaniasis in the Americas. The *Viannia* subgenus causes exclusively tegumentar leishmaniasis in the Americas. Thus, in our data description, in order to evaluate the chitinase gene from the most representative subgenera of *Leishmania* species with clinical importance, were included the chitinase encoding gene from *L. amazonensis* and *L. mexicana* (subgenus *Leishmania*) which are endemic in America; *L. infantum* (subgenus *Leishmania*) original from Old World and disseminated in Americas; and *L. braziliensis*(subgenus *Viannia*).

**Fig. 1:**
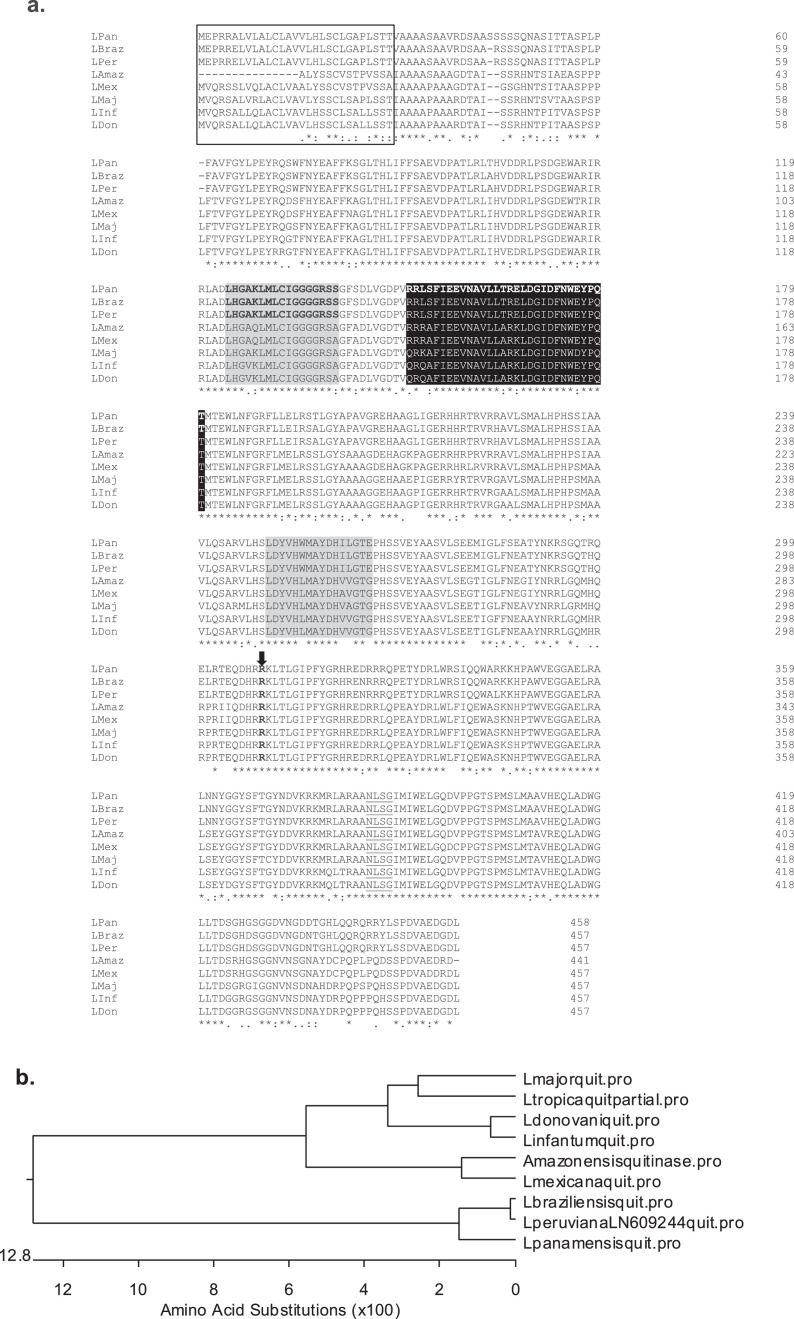
M**ultiple *Leishmania* species chitinase amino acid sequence alignment by MUSCLE. a.** Signal peptide – box; conserved N-glycosylation signal – underlined; substrate recognition substrate – grey shadow; catalytic domain – black shadow; kinase cAMP dependent domain – black arrow. **b.** Dendogram of amino acid chitinase sequence distance analysis showing *Leishmania* species clustering.

*In silico* analysis of the *Leishmania* species chitinase gene data, retrieved from Genbank, classified the chitinase gene in the glycosil-hydrolase 18 (GH18) family.The gene is localized in chromosome 16, and it is encoded by a single copy gene, indicating a high inter-genera identity in all important putative domains and post-translational modifications (Fig. 1). An estimation of evolutionary distance, when using chitinase amino acid sequence, clustered *Leishmania* species according to their evolutionary correlation (Fig. 1) [2]. Prediction of physicochemical characteristics in primary *Leishmania* species chitinase amino acid sequences, lacking signal exportation peptides, are described in Table 1.

**Fig. 2:**
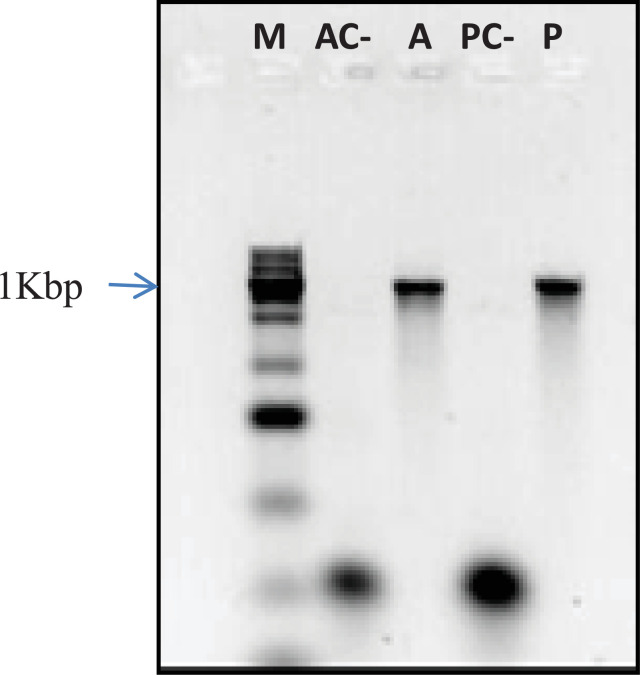
**1% agarose gel electrophoresis to analyse the product of *L. infantum chagasi* chitinase encoding gene expression in amastigotes (A) and promastigotes (P) evaluated by RT/PCR.** AC- and PC- correspond to amastigotes and promastigotes negative controls which have RNA treated with Rnase before cDNA synthesis, respectively; M - 1Kbp ladder (Sinapse).

Reverse transcription followed by PCR (RT-PCR) was performed to amplify a 1 kbp fragment corresponding to the *L. infantum* chitinase, obtained from amastigotes and promastigotes. It confirmed the enzyme expression in the parasite developmental stages in vertebrate and invertebrate hosts (Fig. 2).

With the exception of *L. amazonensis,* for which only a part of the 5’ end was available at Genbank, when obtaining the complete chitinases coding sequences from *L. braziliensis, L. Infantum,* and *L. mexicana*, oligonucleotides complementary to the 5’ and 3’ ends of the specific chitinase encoding gene (Table 2) were used in PCR reactions with high fidelity enzymes. A sense oligonucleotide complementary to the 5’ end in *L. mexicana*, in combination with an antisense oligonucleotide corresponding to the available specific 3’ end sequence, were used for amplifying the *L. amazonensis* chitinase gene sequence without the signal peptide encoding region. The 1287 bp PCR fragment corresponding to the chitinase encoding gene of each *Leishmania* species was cloned into pGEM-T plasmid, whereupon specificity was confirmed according to Sanger sequencing [3] and Basic Local Alignment Search Tool (BLAST) analysis [4]. The new *L. amazonensis* chitinase gene sequence was deposited in Genbank [Genbank:MG869127].

In order to express the recombinant chitinase of each *Leishmania* species included in the study in a prokaryotic system, the 1287 bp chitinase gene fragment, cloned into pGEM-T, was subcloned into pET28a plasmids and after sequence confirmation, recombinant plasmids were named pET28aLachit, pET28aLbchit, pET28aLichit and pET28aLmChit, for *L. amazonensis, L. braziliensis, L. infantum* and *L. mexicana* chitinase constructions, respectilvely. Cloning into pET28a resulted in an addition of six residues of histidine (6xhis-tag) and a thrombin protease peptide signal at the N-terminus of the resulting recombinant protein, used for purification through nickel affinity chromatography and for the removal of the 6xhis-tag if necessary, respectively.

**Fig. 3:**
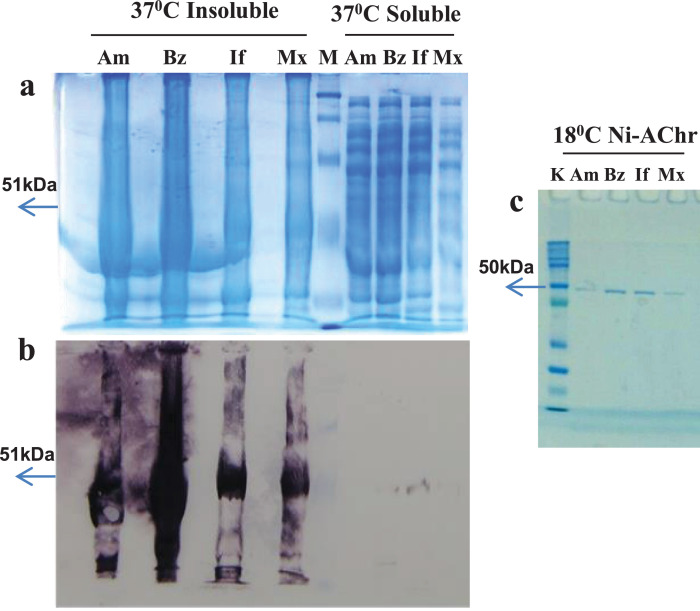
**Expression of *L. amazonensis* (Am), *L. braziliensis* (Bz), *L. infantum* (If) and *L. mexicana* (Mx) recombinant chitinase produced in the *E. coli* strain BL21 (D3) at 37°C and 18°C. a.** SDS-PAGE stained with PageBlue™, a comassie based gel staining solution purchased from Uniscience**; b.** Western blotting with anti-histidine antibody detected by colorimetric staining with BCIP/NBT, after hybridization with second antibody labeled with alkaline phosphatase. **c.** Recombinant protein purified by nickel affinity chromatography from soluble fraction of *E. coli* strain BL21 (D3), induced for 16hs with 1mM IPTG at 18°C; **M and K –** Broad range and kaleidoscope pre-stained protein markers from BioRad; **Insoluble –** pellet of bacteria after lyse; **Soluble** – soluble fraction of bacteria after lyse; **Ni-AChr** – nickel affinity chromatography**.**

Recombinant chitinase protein from each *Leishmania* species, produced in *Eschericchia coli*, when expressed at 37 °C are insoluble, whereas at 18 °C and overnight, are partially soluble, and thus, susceptible to purification by nickel affinity chromatography (Fig. 3). SDS-PAGE and Western blotting analysis, using a mouse raised monoclonal anti-histidine antibody purchased from Sigma, revealed a chitinase recombinant protein from *Leishmania* species weighting approximately 50 KDa (Fig. 3B), including the 6xhis-tag and the thrombin recognition amino acid sequences derived from the vector. Comparison of *Leishmania* species primary chitinase sequences without signal exportation peptide, with their corresponding recombinant forms produced in *E. coli* showed an increase in the isoelectric point and in the protein charge at pH 7.0 of approximately +0.3 and +2.0, respectively (Table 1). The *E. coli* recombinant protein yield after the purification by nickel affinity chromatography was 200 ug/liter of the initial bacterial culture. The value was comparable among all *Leishmania* species chitinases. After the purification, recombinant proteins precipitated by different buffers (20 mM and 150 mM phosphate buffer pH 7.4, with and without 50 mM NaCl and/or 10% glycerol) and under distinct temperature conditions (room temperature, 4 °C, −20 °C and −80 °C), revealing a high structural instability, which circumvented their biochemical characterization.

**Table 1:** *In silico* predicted physicochemical characteristics of recombinant *Leishmania* species chitinase proteins according to Lasergene

	Chitinase sps				Prokaryote – N-his				Eukaryote – C-his				Eukaryote – N-His/C-His			
	IP	Ch	MW	aa	IP	Ch	MW	aa	IP	Ch	MW	aa	IP	Ch	MW	aa
***L. amz***	6,72	-2,11	47,5	427	7,00	+0,06	49,9	448	6,99	-0,10	52,4	468	7,23	+2,06	54,3	485
***L.brz***	7,19	+1,10	48,1	429	7,54	+3,26	50,4	450	7,52	+3,09	52,9	469	7,88	+5,26	54,8	486
***L.inf***	6,51	-4,00	47,5	429	6,80	-1,90	49,8	450	6,78	-2,00	52,3	469	7,00	+0,09	54,2	486
***L.mex***	6,55	-3,90	47,5	429	6,80	-1,77	49,8	450	6,80	-1,94	52,8	469	7,00	+0,22	54,2	486

IP – Isoeletric point; Ch – Charge at pH 7.0; MW – Molecular weight; aa – number of amino acids; ***L. amz****– Leishmania amazonensis;****L.brz****– Leishmania braziliensis;****L. inf****– Leishmania infantum****; L. mex****– Leishmania mexicana*; **chitinase sps** – chitinase without signal exportation peptide; **Prokaryote** – recombinant chitinase cloned in pET-28a for expression in bacteria; **Eukaryote** - recombinant chitinase cloned in HBM-Topo for baculovirus production and expression in Sf9 cells; **N-his and C-his** – 6xhis tag at recombinant protein N-terminus and C-terminus, respectively.

**Table 2:** Description of the oligonucleotides used in the study

Name	Sequence (5’-3’)	Conditions	Specificity/Amplicon (bp)
LquitExp_^a^sps_brazil_fow	ACGGTGGCGGCTGCTGCGTCTGCA	94 °C 3’/40 × 94 °C 1’-54 °C 30"’-72 °C 1′/72 °C 7’	*L. braziliensis /* 1.374
LquitExp_sps_brazil_rev	TAGATCACCGTCCTCG		
LquitExp_sps_infant_fow	ACTATAGCCGCTGCTGCTCCTGCA	94 °C 3’/40 × 94 °C 1’-62 °C 30"’-72 °C 1′/72 °C 7’	*L. infantum chagasi /* 1.374
LquitExp_sps_infant_rev	TAGATCACCGTCCTCAGCCACATC		
LquitExp_sps_mex_fow	GCTATAGCCGCTGCTGCTCCTGCA	94 °C 3’/40 × 94 °C 1’-65 °C 30"’-72 °C 1′/72 °C 7’	*L. mexicana /* 1.374
LquitExp_sps_mex_rev	TAGATCGCGGTCCTCAGCCACGTC		
LquitExp_sps_amz_fow	GCTATAGCCGCTGCTGCTTCTGCA	94 °C 3’/40 × 94 °C 1’-65 °C 30"’-72 °C 1′/72 °C 7’	*L. amazonensis /* 1.374
LquitExp_sps_mex_rev	TAGATCGCGGTCCTCAGCCACGTC		
LCQuitFow	GCTGCCTGAGCGCTCT	94 °C 3’/40 × 94 °C 1’-65 °C 30"’-72 °C 1′/72 °C 7’	*L. infantum*/ 1000 [12]
LCQuitRev	CTCCCTCGACCCATGTT		
T7F	TTAATACGACTCACTATAGGGGAATTG	94 °C 3’/40 × 94 °C 1’-48 °C 30"’-72 °C 1′/72 °C 7’	pET28a vector / 1419
T7R	GCTAGTTATTGCTCAGCGGTGG		
pET28hisF	CATCATCATCATCATCACAGCAGCGGC	94 °C 3’/40 × 94 °C 1’-65 °C 30"’-72 °C 1′/72 °C 7’	*Leishmania* sp chitinase cloned into pET28a / 1419
PolyhedrinF	AAATGATAACCATCTCGC	94 °C 3’/40 × 94 °C 30’’-50 °C 30’’-72 °C 1’/72 °C 7’	HBM-TOPO vector/ 300 plus size of the insert
SV40R	GGTATGGCTGATTATGATC		
M13F	CCCAGTCACGACGTTGTAAAA	94 °C 3’/40 × 94 °C 30’’-55 °C 30’’-72 °C 1’/72 °C 7’	Bacmid / 2300 plus size of the insert
M13R	AGCGGATAACAATTTCACACAGG		

a sps- No signal peptide.

In order to obtain the recombinant chitinase with post-translational modifications and precise folding, all the four recombinant chitinases from each *Leishmania* species were expressed in insect cells using baculoviruses produced according to the *Bac-to-Bac* system, purchased from Thermo Fisher Scientific. Initial construction included a *Tobacco Etch Virus* protease signal sequence followed by a 6xhis-tag at the C-terminus of recombinant proteins. SDS-PAGE and Western blotting (anti-histidine antibody) revealed the recombinant chitinase with approximately 52 KDa, analogous to the *L. infantum* and *L. mexicana* chitinases, after 48hs of baculoviruses infection, in the insoluble phase of the culture. Heterologous proteins were not exported to the extracellular medium. According to the specifications of the commercial expression system used it was not expected. *L. amazonensis* and *L. braziliensis* recombinant chitinases were not observed at any time or fraction of the culture (data not shown). The corresponding *L. amazonensis* and *L. braziliensis* chitinase mRNA was visualized by RT-PCR after 24hs of Sf9 baculoviruses infection (data not shown).

**Fig. 4:**
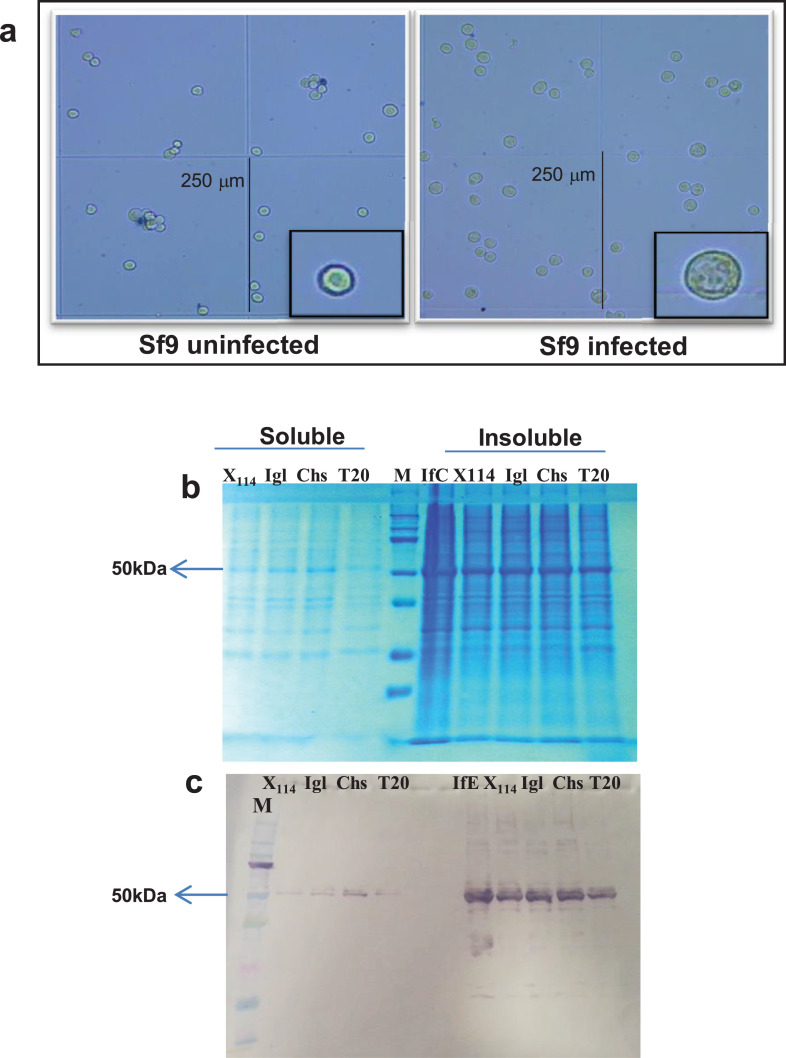
**Detergent solubilization assay of *L. infantum* recombinat chitinase expressed in *Spodoptera frugiperda* (Sf9) ovarian cells infected with *L. infantum* chitinase baculovirus. a.** Sf9 and Sf9 cells infected with *L. infantum* chitinase recombinant baculovirus photographed under Neubauer hemocytometer. **b.** SDS-PAGE stained with PageBlue™; **c.** Western blotting with the anti-histidine antibody revealed with BCIP/NBT colorimetric reagents for alkaline phosphatase. **M:** Spectra Multicolor Broad Range Ladder (Thermofisher). **IfC –** SF9 cells infected with *L. infantum* chitinase recombinant baculoviruses; **X_114_- 1% Triton X114; Igl – 1% Igepal; Chs – 1% CHAPS; T20 – 1% Tween20. Soluble/Insoluble:** Soluble and Insoluble fractions of Sf9 cells after detergent and 1% CHAPS treatment.

The various experimental conditions employed for dissolving the *Leishmania* chitinase from insect cells included membrane-cell disruption by ultrasound, and thaw/freezing cycles with dry ice and liquid nitrogen in the presence of protease inhibitors and detergents [CHAPS (1%), IGepal (1%), Triton X-114 (2%) and Tween 20 (1%)] (Fig. 4). *Leishmania* genus recombinant chitinases were partially soluble after sonication with all the detergents employed. The best results were obtained with CHAPS 1% or Tween 20 1% in PBS (Fig. 4). The soluble fraction containing recombinant chitinases underwent purification by nickel affinity chromatography, gel filtration, and ion exchange chromatography. However, *Leishmania* recombinant chitinases did not bind to the nickel affinity chromatographic column; during gel filtration size purification, the protein was present in all fractions, thereby indicating the presence of protein oligomers of different sizes. Thus, it was impossible to obtain pure recombinant proteins through ion exchange chromatography (data not shown).

In order to circumvent the absence of *L. amazonensis* and *L. brasiliensis* recombinant proteins, and the insolubility of recombinant chitinase from *L. infantum chagasi* and *L. mexicana*, new constructions with 6xhis-tag at the N-terminals were made. Chitinases from all the four *Leishmania* species were included in the study. Thence, recombinant chitinase from all the four species was produced at high concentrations, thereby demonstrating that 6xhis-tag at N-terminals conferred more stability to recombinant proteins. However, after several attempts to purify *Leishmania sp* chitinase from insect cells, only a small fraction (less than 10%) could be purified by nickel affinity chromatography, and after removal of imidazole, which is used in the step of dissociation of the his-tagged protein from the chromatographic column, the recombinant proteins precipitated and were insoluble.

The results from dot blot serological testing, using denatured proteins obtained from bacteria and insect cell membrane with recombinant chitinases as antigen, were inconclusive (data not shown).

The GH18 chitinase from *Leishmania* was elected as an important target for leishmaniasis serological diagnostic, considering its predicted biochemical characteristics, including exportation signal to extracellular compartment and biological importance [5, 6], associated to its absence in the *Trypanosoma* genus (Table 1). However, prokaryotic heterologous expression produced insoluble protein, impairing its purification. Thus, when considering a successful expression of the *L. major* GP63, a glycosylated membrane protein, in insect cells using recombinant baculovirus [7], and a predicted glycosylation site in *Leishmania* chitinase, different baculoviruses constructions of the four *Leishmania* species chitinase were performed using the *Bac-to-Bac* insect cell expression system. Predicted physicochemical characteristics of the resulting recombinant proteins revealed differences in isoelectric point and charge (Table 1), which should guarantee some molecular stable and soluble recombinant form of the protein. Nevertheless, all constructions were unable to result in solvable protein, even after several chemical and physical treatment attempts. These results suggest a *Leishmania* species chitinase intrinsic molecular characteristic associated to its insolubility and post-translational signals, making it incompatible with insect cells organelles machinery, resulting in accumulation of unfolded recombinant protein in cellular compartments. Insolubility of recombinant forms of *Leishmania* species chitinase, independently of the differential physicochemical characteristics, strongly suggests the occurrence of a molecular specific feature necessary to precise *Leishmania* chitinase folding, such as chaperones. Thus, non-pathogenic *Leishmania* species should be considered as the models organisms of choice for protein expression of *Leishmania* specific genes, such as chitinase [8].

## 2 Experimental Design, Materials and Methods

### 2.1 *Leishmania* species chitinase encoding gene and protein *in silico* evaluation

*Leishmania* species chitinase encoding sequences were obtained from Genbank. The *L. mexicana* chitinase gene was employed in BLAST research [4]. Complete and partial sequences available for *L. amazonensis* [AY518257.1], *L. braziliensis* [FR798990.1], *L. donovani* (XM_003859718.1], *L. infantum* [FR796448.1], *L. major* [FR796412.1], *L. mexicana* [FR799569.1], *L. panamensis* [CP009385.1], *L. peruviana* [LN609244.1], *L. tropica* [AY518258.1] chitinase were used for *in silico* comparative analysis when applying the multiple sequence alignment method – MUSCLE (Fig. 1) [9]. Signal exportation peptide and classification features of *Leishmania* chitinase in the GH18 family were presumed using previous studies of *L. donovani* [10] and *L. mexicana* [6]. Prediction of *Leishmania* chitinase post-translational modifications and domains annotations were investigated using ScanProsite (Figure 1) [11].

### 2.2 Native chitinase RNA expression

RNA from *L. infantum i* amastigotes, of an infected mouse and promastigotes cultivated in a M199 medium at 28 °C, was extracted using a RNA minikit (Qiagen) according to manufacturer's instructions. Chitinase expression was verified by RT-PCR with MMLV reverse transcriptase, purchased from Thermo Fisher Scientific, using 5uL of total RNA treated with DNAse and 50uM of LquitExp_sps_infant_rev. PCR was applied to amplify a 1 kbp *Leishmania* chitinase fragment (Table 2) [12]. As negative control, RNA samples were submitted to RNAse prior cDNA synthesis.

### 2.3 Cloning of *Leishmania* species chitinase

The chitinase encoding gene without signal exportation peptide was amplified from genomic DNA of *L. amazonensis, L. brasiliensis, L. infantum,* and *L. mexicana* obtained from the *Leishmania* Collection of the Instituto Oswaldo Cruz *-*CLIOC *-* Rio de Janeiro*,* Brazil (Reference IOCL 0575, 0566, 0579 and 0561, respectively). PCR amplification was performed using a *Plantinum Taq DNA polymerase High Fidelity* (Thermo Fisher Scientific) with specific oligonucleotides (Table 2) cloned into a pGEM-T Easy vector system (Promega). Recombinant plasmids denominated pGEMT-La, Lb, Li and LmChit, for chitinase obtained from *L. amazonensis, L. braziliensis, L. infantum* and *L. mexicana*, respectively, were selected by PCR with oligonucleotides M13F and M13R (Table 2), and confirmed by Sanger sequencing and alignment with reference *Leishmania* species chitinase sequences available in GenBank.

### 2.4 Prokaryotic expression

Oligonucleotides were designed to include either the N*deI* or N*heI* and H*indIII* enzyme restriction sites at the 5′ and 3′ ends, respectively, of a 1287 bp chitinase encoding gene PCR fragment amplified with *Platinum* Taq High Fidelity DNA polymerase (Thermo Fisher Scientific) (Table 2) on pGEMT chitinase clones. The obtained chitinase amplicons were ligated with T4 DNA ligase to the corresponding restrictions sites of pET28a (Novagen) and transformed into *E. coli* One Shot Match 1TM-T1R chemically competent cells (Thermo Fisher Scientific). Specificity of the subsequent recombinant pET28a-La, Lb, Li and LmChit plasmids to chitinase genes from *L. amazonensis, L. braziliensis, L. infantum* and *L. mexicana*, respectively, was confirmed through Sanger sequencing. Recombinant chitinase expression was achieved in competent *E. coli* BL21 (DE3) cells, cultivated in a LB medium containing 50 µg/mL of kanamycin at a 600 nm Optical Density of 0,6 (37 °C), induced by 0,1 M isopropyl-βD-thiogalactopyranoside (IPTG) and grown during four hours at 37 °C or overnight at 18 °C in a shaker (250 rpm). One liter of bacterial pellet culture, obtained after centrifugation at 10.000x*g* for 10 min at 4 °C, was disrupted after incubation for 15 min at room temperature with 20 mL of buffer A (20 mM sodium phosphate pH 7.4, 1 mM EDTA, 50 mM Dextrose and lysozyme (4 mg/mL)), and the addition of 20 mL of buffer B (20 mM sodium phosphate pH 7.4, 1 mM EDTA, 50 mM potassium chloride and 0,5% Tween 20). Following centrifugation at 10.000x*g* for 20 min at 4 °C, and after equilibration with 10 column volumes of 20 mM sodium phosphate pH 7.4 and 20 mM imidazole, soluble protein extracts were applied to a nickel gravity flow chromatography column (1 mL). Subsequently, the column was washed with 10 column volumes of 20 mM sodium phosphate pH 7.4 and 50 mM imidazole whereupon all the recombinant proteins were eluted with 2 mL of 20 mM sodium phosphate pH 7.4 and 150 mM imidazole pH 7.4. To obtain recombinant chitinase free of imidazole, these were submitted to size exclusion chromatography with Sephadex 100 in 20 mM sodium phosphate pH 7.4. Protein fractions were evaluated with 12% SDS-PAGE and Western blot on nitrocellulose membranes, using a mouse raised monoclonal anti-histidine antibody (Sigma). After hybridization with a rabbit anti-mouse IgG secondary antibody containing alkaline phosphatase, 6xhis-tag proteins were visualized by adding colorimetric substrate nitro-blue tetrazolium (NBT) and 5-bromo-4chloro-3’-indolyphosphate (BCIP).

### 2.5 Eukaryotic system

The eukaryotic method for expressing the *Leishmania* species chitinase encoding gene without a signal peptide was the *Bac-to-Bac HBM-TOPO* expression system, purchased from Thermo Fisher Scientific, and applied according to manufacturer instructions. Two constructions of each *Leishmania* species chitinase were performed, one with a his-tag at C-terminus, and the other with the tag at the N and C-terminus. *Leishmania* species chitinase encoding genes were amplified by PCR using pET28a-La, Lb, Li and LmChit as targets, *Platinum* Taq High Fidelity DNA polymerase and oligonucleotides, under the conditions described in Table 2. Before the production of recombinant baculoviruses in *Spodoptera frugiperda* (Sf9) ovarian cells, cultivated in SF900III or ESF921 medium at 28 °C, all recombinant clones were checked by Sanger sequencing. After transfection, baculovirus (BV) infection cytopathic effects were monitored daily for five days. The titer of the obtained recombinant baculoviruses was amplified by two subsequent passages (titer of 10^7^ BV/mL) in Sf9 cells. Recombinant chitinase baculoviruses from *L. amazonensis, L. braziliensis, L. infantum* and *L. mexicana*, with his-tags at C-terminus were denominated BV-La, Lb, Li and LmChit; and those with the tag at the N and C-terminus, NBV-La, Lb, Li and LmChit.

Expression of the recombinant *Leishmania* species chitinase was obtained in Sf9 and High Five (Thermo Fisher Scientific) insect cells. Once reaching 50–70% of the cytopathic effect (72–96 h post infection), infected cells were collected and then centrifuged at 10,000x*g* at 4 °C for 10 min. The supernatant was kept at 4 °C until use. The remaining cell-pellets were incubated in a digestion buffer solution (10 mM phosphate buffer pH 8.0, 1 mM MgCl_2_, 150 mM NaCl, 0,5mM EGTA, 0,5 mM DTT, 10% Glicerol, 2% Triton X-100, 1x Halt Protease Inhibitor Cocktail, EDTA Free) (Thermo Fisher Scientific),. Following six cycles of chilling and frozen and 10 min of centrifugation at 10,000x*g* and 4 °C, the insoluble fraction was separated. Alternatively, cell pellets from a 100 mL culture were diluted in 3 mL of 20 mM phosphate buffer pH 7.4 and various detergents, viz., CHAPS (1%), IGepal (1%), Triton X-114 (2%) and Tween 20 (1%), with subsequent disruption by sonication. Soluble and insoluble protein fractions were submitted to affinity chromatography in a nickel-charged resin using the Ni-NTA fast-speed protein purification kit (Qiagen), according to manufacturer's instructions. Recombinant protein preparations were analyzed by SDS-PAGE and Western blotting on nitrocellulose membrane (GeHealthcare), with a mouse C-terminus anti-his antibody (Thermo Fisher Scientific).

## Ethical Statement

RNA from animals used in this study was donated by Dr. Luis Felipe D. Passero and manipulation ethical aspects were published [13] in accordance with the recommendations from the Guide for the Care and Use of Laboratory Animals of the Brazilian National Council of Animal Experimentation (http://www.cobea.org.br). Source of sera from human, used as controls in serology tests, was described in other study of our research group [14].

## Declaration of Competing Interest

The authors declare that they have no known competing financial interests or personal relationships which have, or could be perceived to have, influenced the work reported in this article.

## Appendix Supplementary materials

Image, application 1
